# Identifying threshold sizes for enlarged abdominal lymph nodes in different age ranges from about 200,000 individual’s data

**DOI:** 10.1038/s41598-021-81339-9

**Published:** 2021-01-19

**Authors:** Lili He, Yinghua Sun, Guoying Huang

**Affiliations:** 1 Department of Ultrasound, Children’s Hospital of Fudan University, Shanghai, 201102 People’s Republic of China; 2 Cardiovascular Center, Children’s Hospital of Fudan University, Shanghai, 201102 People’s Republic of China

**Keywords:** Diseases, Signs and symptoms

## Abstract

The threshold size for enlarged abdominal lymph nodes (E-ALNs), a common pediatric disorder, has yet to be standardized. According to the maximum short-axis diameter, this study divided ALNs into Grade A (≥ 10 mm), Grade B (8–10 mm), Grade C (5–8 mm), and Grade D (< 5 mm, normal). To identify the threshold size for E-ALNs, the prevalence of each grade was compared between asymptomatic individuals and symptomatic (e.g., abdominal pain) individuals without other diseases (e.g., appendicitis) that could explain the symptoms for different ages using data from > 200,000 individuals. The results showed the following: (1) For ages 1–3 years, the recommended threshold size is 8 mm, as the differences in the prevalence between the two groups were nonsignificant for Grade C but significant (p < 0.05) for both Grades A and B. (2) For ages 3–14 years, the recommended threshold size is 5 mm, as the differences between the two groups were significant (p < 0.05) for Grades A, B, and C. (3) The prevalence of Grades A, B, and C was very low for ages 0–1 years and high for ages 1–6 years. (4) The prevalence for males was generally higher than that for females for Grades A and B.

## Introduction

Enlarged abdominal lymph nodes (E-ALNs) in children, or what some researchers (mostly radiologists) refer to as “mesenteric lymphadenitis (ML)”, is a prevalent pediatric disorder. ML was first described in 1926^1^ and often manifests with gastrointestinal symptoms such as abdominal pain, diarrhea, nausea, and vomiting, as well as other symptoms such as fever and upper respiratory tract infections. However, children with these abdominal symptoms may also be diagnosed with other diseases after examination, such as appendicitis, intussusception, lithiasis, and ovarian torsion. In fact, sonographers can often identify E-ALNs in asymptomatic children, symptomatic individuals without other diseases that could explain the symptoms, and symptomatic patients with other diseases, as mentioned above. Nevertheless, the size of the lymph nodes may be different between symptomatic and asymptomatic children^2^. ML was first radiologically defined as a cluster of lymph nodes with a short-axis diameter (SAD) ≥ 5 mm^3^ in 1993. Subsequently, different opinions were proposed, and there has been a large discrepancy in the recommended threshold size for E-ALNs^4–7^. To date, the prevalence and size criteria for E-ALNs in children remain controversial. In recent years, reports of ML. or E-ALNs. have been rare^8^. Published studies are mainly based on very limited asymptomatic populations (usually fewer than 1000 individuals), and consequently, the prevalence of E-ALNs varies greatly^6,7^. Moreover, as admitted by the investigators themselves, some studies failed to include an asymptomatic control group, which may affect the accuracy of their results^9^.

To fill this literature gap, using the data from more than 200,000 individuals, this study calculates and compares the prevalence of different grades of ALNs among three groups consisting of male and female subjects in different age ranges: group 1, the asymptomatic group (AS); group 2, symptomatic individuals without other diseases that could explain the symptoms (Sw/ood); and group 3, symptomatic patients diagnosed with other diseases after examination (Swod). Then, the threshold size for E-ALNs in children and adolescents is identified. Finally, the age and sex prevalence distributions are analyzed and summarized.

## Results

### Statistical characteristics of 206,775 subjects

**Figure 1:**
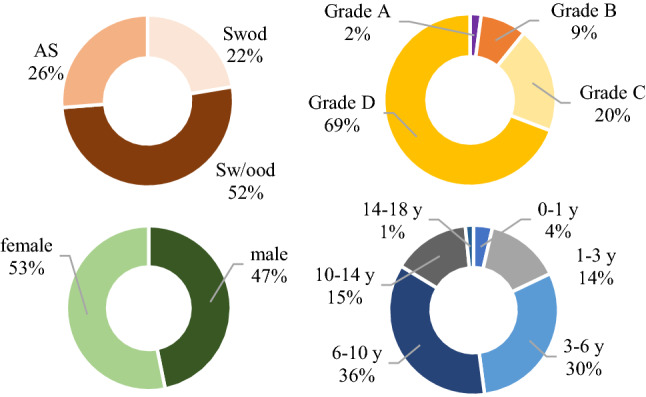
Clinical symptoms and diagnoses, ALN grades, sexes, and ages of the 206,775 subjects.

The demographic and clinical characteristics of the subjects are shown in Fig. 1. A total of 206,775 subjects were enrolled, including 96,756 male subjects and 110,019 female subjects. The subjects ranged from 1 day to 18 years old, with an average age of 6.66 ± 3.44 years.

Among the subjects, 54,232 were in the AS group, including 12,595 male and 41,637 female individuals, with an average age of 8.95 ± 2.30 years; 106,311 were in the Sw/ood group, including 57,528 male and 48,783 female subjects, with an average age of 5.77 ± 3.00 years; and 46,232 were in the Swod group, including 26,633 male and 19,599 female patients, with an average age of 6.02 ± 4.21 years.

### Number and prevalence of subjects

As groups 1–3 were significantly different (p < 0.05) in terms of age and sex distributions, each group was divided into 6 age ranges and 2 sexes, forming 12 age-sex subgroups that were subsequently subjected to pairwise intergroup comparisons. The number of subjects and the prevalence in each subgroup are presented in Table 1.

**Table 1:** Number and prevalence of subjects in different age- and sex-grade subgroups of the AS, Sw/ood, and Swod groups.

Age (years)	Group	Female						Male					
		Grade A	Grade B	Grade C	Grade D	Total	P-value	Grade A	Grade B	Grade C	Grade D	Total	P-value
0–1	AS	0 (0.00%)	0 (0.00%)	15 (10.34%)	130 (89.66%)	145	0.067^a^ < 0.001^b^	0 (0.00%)	1 (2.44%)	2 (4.88%)	38 (92.68%)	41	0.225^a^ 0.057^b^
	Sw/ood	3 (0.37%)	21 (2.58%)	54 (6.64%)	735 (90.41%)	813		5 (0.45%)	27 (2.44%)	70 (6.32%)	1005 (90.79%)	1107	
	Swod	10 (0.45%)	27 (1.21%)	82 (3.69%)	2106 (94.65%)	2225		23 (0.74%)	70 (2.25%)	137 (4.41%)	2876 (92.59%)	3106	
1–3	AS	5 (0.78%)	46 (7.15%)	137 (21.31%)	455 (70.76%)	643	< 0.001^a^ < 0.001^b^	0 (0.00%)	15 (8.77%)	38 (22.22%)	118 (69.01%)	171	< 0.001^a^ < 0.001^b^
	Sw/ood	266 (3.08%)	1077 (12.48%)	2006 (23.25%)	5281 (61.19%)	8630		483 (4.38%)	1493 (13.54%)	2407 (21.83%)	6644 (60.25%)	11,027	
	Swod	99 (2.54%)	341 (8.74%)	843 (21.61%)	2618 (67.11%)	3901		225 (4.29%)	570 (10.87%)	1113 (21.22%)	3338 (63.62%)	5246	
3–6	AS	18 (0.57%)	130 (4.14%)	590 (18.80%)	2401 (76.49%)	3139	< 0.001^a^ < 0.001^b^	22 (1.36%)	89 (5.52%)	328 (20.33%)	1174 (72.78%)	1613	< 0.001^a^ < 0.001^b^
	Sw/ood	489 (2.36%)	2530 (12.20%)	6192 (29.87%)	11,522 (55.57%)	20,733		962 (3.94%)	3617 (14.82%)	6780 (27.78%)	13,047 (53.46%)	24,406	
	Swod	88 (1.72%)	432 (8.45%)	1122 (21.95%)	3470 (67.88%)	5112		179 (2.55%)	741 (10.56%)	1380 (19.66%)	4720 (67.24%)	7020	
6–10	AS	110 (0.36%)	864 (2.84%)	3937 (12.93%)	25,527 (83.87%)	30,438	< 0.001^a^ < 0.001^b^	37 (1.02%)	178 (4.90%)	490 (13.48%)	2930 (80.61%)	3635	< 0.001^a^ < 0.001^b^
	Sw/ood	227 (1.66%)	1423 (10.40%)	3952 (28.88%)	8084 (59.07%)	13,686		495 (3.21%)	1989 (12.89%)	3827 (24.80%)	9119 (59.10%)	15,430	
	Swod	55 (1.17%)	258 (5.50%)	806 (17.17%)	3574 (76.16%)	4693		132 (2.16%)	499 (8.16%)	909 (14.87%)	4573 (74.81%)	6113	
10–14	AS	13 (0.18%)	115 (1.61%)	634 (8.90%)	6360 (89.30%)	7122	< 0.001^a^ < 0.001^b^	54 (0.81%)	269 (4.03%)	769 (11.51%)	5587 (83.65%)	6679	< 0.001^a^ < 0.001^b^
	Sw/ood	59 (1.34%)	283 (6.41%)	812 (18.40%)	3260 (73.86%)	4414		174 (3.39%)	494 (9.62%)	962 (18.74%)	3504 (68.25%)	5134	
	Swod	18 (0.62%)	102 (3.50%)	280 (9.62%)	2512 (86.26%)	2912		67 (1.58%)	216 (5.10%)	436 (10.29%)	3517 (83.03%)	4236	
14–18	AS	0 (0.00%)	0 (0.00%)	9 (6.00%)	141 (94.00%)	150	0.176^a^ 0.089^b^	3 (0.66%)	15 (3.29%)	46 (10.09%)	392 (85.96%)	456	0.190^a^ 0.144^b^
	Sw/ood	5 (0.99%)	11 (2.17%)	37 (7.29%)	454 (89.55%)	507		5 (1.18%)	23 (5.42%)	53 (12.5%)	343 (80.90%)	424	
	Swod	4 (0.53%)	10 (1.32%)	35 (4.63%)	707 (93.52%)	756		8 (0.88%)	38 (4.17%)	83 (9.10%)	783 (85.86%)	912	
All ages		1469 (1.34%)	7670 (6.97%)	21,543 (19.58%)	79,337 (72.11%)	110,019		2874 (2.97%)	10,344 (10.69%)	19,830 (20.49%)	63,708 (65.84%)	96,756	

The superscript a indicates P-values for the comparison between the AS and Sw/ood groups, while b indicates P-values for the comparison between the Sw/ood and Swod groups.

The analysis was performed to assess the differences in prevalence among the three groups. The results showed that there were no statistically significant differences in the prevalence between 0 and 1-year-old AS and Sw/ood individuals of the same sex (p > 0.05) and between 14 and 18-year-old AS and Sw/ood individuals of the same sex (p > 0.05). For the subgroups spanning 1–14 years, there were statistically significant differences in the prevalence among the same-sex subgroups (p < 0.001), indicative of a different distribution of abdominal lymph nodes among the three groups of 1–14-year-old subjects.

For ages 1–3 years, there were statistically significant differences (p < 0.05) in the prevalence of Grades A and B between the AS and Sw/ood groups for both females and males. However, the differences in the prevalence of Grade C between the AS group and the Sw/ood group were insignificant for both females (p = 0.261 for a prevalence of 23.25% vs. 21.31%) and males (p = 0.902 for a prevalence of 21.83% vs. 22.22%). Thus, Grade C could be excluded from the range of abnormally sized abdominal lymph nodes for children aged 1–3 years. As a result, the recommended threshold size for E-ALNs at ages 1–3 years is 8 mm (see Table 2).

**Table 2:** Recommended threshold sizes for E-ALNs in different age ranges.

Age (years)	Grade A (SAD: ≥ 10 mm)	Grade B (SAD: 8–10 mm)	Grade C (SAD: 5–8 mm)	Threshold size
1–3	p < 0.05	p < 0.05	p = 0.902 for male, p = 0.261 for female	≥ 8 mm
3–14	p < 0.05	p < 0.05	p < 0.05	≥ 5 mm

For ages 3–14 years, there were statistically significant differences (p < 0.05) in the prevalence of Grades A, B and C between the AS group and the Sw/ood group for all children in the same age range. As Grade D is universally accepted as the normal group according to the radiological definition of ML^3^, the recommended threshold size for E-ALNs at ages 3–14 years is 5 mm (see Table 2).

**Figure 2:**
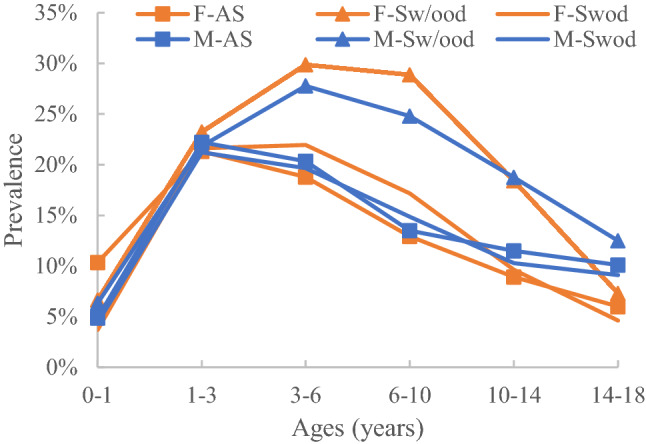
Prevalence of Grade C by subgroup.

**Figure 3:**
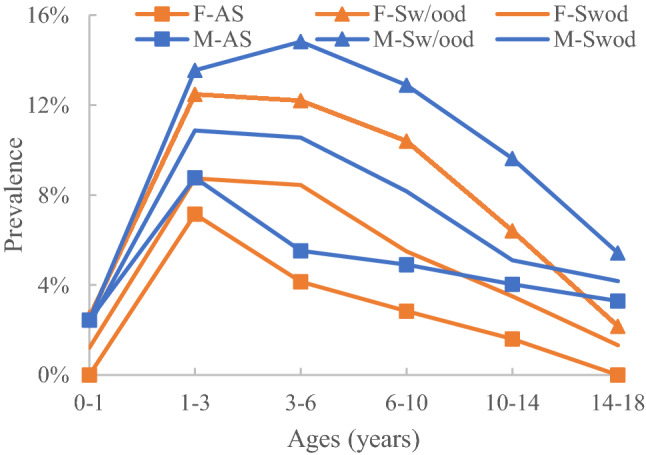
Prevalence of Grade B by subgroup.

**Figure 4:**
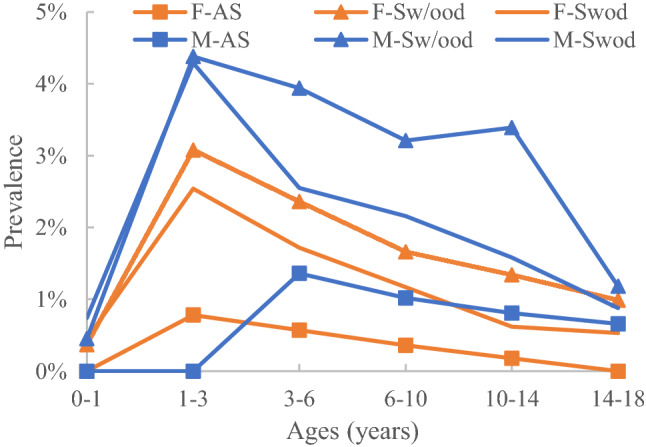
Prevalence of Grade A by subgroup.

The distribution characteristics of Grades A, B, and C by age and sex in the AS, Sw/ood, and Swod groups are shown in Figs. 2–4.

### Distribution characteristics of Grade C

#### Prevalence distribution by age

The prevalence was low for all six subgroups between the ages of 0–1 years but rapidly increased above 20% in the 1–3 year subgroups. The maximum prevalence was 29.87% in the female Sw/ood 3–6-year subgroup and 27.78% in the male Sw/ood 3–6-year subgroup. For the > 6-year subgroups, the subjects showed a decreasing trend of prevalence with increasing age.

#### Prevalence distribution by sex

In the Sw/ood group, for the 1–10-year age subgroups, the female subgroups had a higher prevalence than their male counterparts (p < 0.05). In the AS group, male subjects aged 10–14 years had a higher prevalence than their female counterparts (p < 0.05).

### Distribution characteristics of Grade B

#### Prevalence distribution by age

The prevalence was very low for ages 0–1 years, with all subgroups at < 3%, but it increased rapidly between the ages of 1–3 years. The prevalence for the male Sw/ood subgroup peaked at 14.82% for 3–6 years, while the prevalence for the other five subgroups gradually decreased with increasing age after the age of 3 years.

#### Prevalence distribution by sex

In all groups, male subjects had a higher prevalence than female subjects in each age range after the age of 1 year (p < 0.05).

### Distribution characteristics of Grade A

#### Prevalence distribution by age

The prevalence was very low for ages 0–1 years, with all subgroups at < 1%. In the Sw/ood group, male and female subjects aged 1–3 years presented the maximum prevalence of 4.38% and 3.08%, respectively.

#### Prevalence distribution by sex

In all groups, male subjects had a higher prevalence than female subjects in each age range (p < 0.05), except for the AS group at ages 1–3 years.

## Discussion

Lymph nodes are important human organs, mainly playing a role in the immune activities of the body. The results of this study showed the following: (1) The overall prevalence of E-ALNs in Sw/ood subjects between the ages of 1–14 years was significantly (p < 0.001) higher than in their AS and Swod counterparts, indicating that E-ALNs are related to gastrointestinal symptoms (e.g., abdominal pain). This may be attributed to the fact that children's immune systems are not yet fully developed. (2) Sw/ood subjects aged 0–1 years and 14–18 years did not differ significantly from their AS counterparts in the prevalence of E-ALNs in general, which was very low in the two age ranges. This may be attributed to the fact that maternal immunity, transferred to subjects < 1 year old, is relatively high, while the immunity of adolescent subjects aged > 14 years is similar to that of adults.

### Threshold sizes for E-ALNs in different age ranges

Abdominal lymph nodes were classified as abnormal with an SAD ≥ 5 mm based on the radiological definition of ML, which was proposed more than 20 years ago^3^. However, this threshold has since been continuously questioned by other researchers. In 2005, Karmazyn et al. analyzed ALN data from 61 children with suspected or confirmed diagnoses of renal calculi, arguing that the threshold should be 8 mm^6^. In 2007, Simanovsky and Hiller analyzed the data from 200 children, arguing that ALN can be considered abnormal only with an SAD > 10 mm^7^. Given this context, ALNs were classified into Grades A-D according to the maximum SAD in this study, and the prevalence of each grade was calculated.

Based on the results in Table 2, it is recommended that the size criterion for E-ALNs be an SAD ≥ 8 mm in children between the ages of 1–3 years and ≥ 5 mm in children and adolescents between the ages of 3–14 years.

### Prevalence of E-ALNs by sex

In regard to sex, some early studies suggested a higher prevalence in male subjects than in female subjects^10,11^. Surprisingly, it was observed in this study that Sw/ood female subjects between 1 and 10 years had a significantly (p < 0.05) higher prevalence of Grade C ALNs than their male counterparts, while the male subjects had a higher prevalence of Grades A and B ALNs. In summary, the male-to-female prevalence ratio is > 2 (2.97–1.34%) for Grade A, approximately 1.5 (10.69–6.97%) for Grade B and close to 1 (20.49–19.58%) for Grade C ALNs. These observations suggest that the greater the degree of ALN enlargement, the greater the sex differences in prevalence. This remains to be further analyzed to better understand the underlying reasons.

### Prevalence of E-ALNs by age

Regarding the age-dependent prevalence, relevant studies are rare, and the existing results are inconsistent. Some studies have shown that the prevalence of E-ALNs decreases with increasing age^10,12^. However, another study argued that the highest prevalence of E-ALNs occurred at 10 years of age^7^. Compared with those studies, our study investigated the entire pediatric age range and revealed that the prevalence was very low before the age of 1 year and increased rapidly afterwards, reaching a maximum between the ages of 1–6 years and gradually decreasing afterwards with increasing age.

### Prevalence of E-ALNs in the Swod group

As mentioned before, E-LANs are often found in some patients diagnosed with other diseases (e.g., appendicitis, intussusception) by ultrasound (US). Moreover, their ALNs are not as numerous or as large as those visualized in patients with primary ML, as argued by Toorenvliet et al.^13^. Our study further explored the prevalence of different grades of ALNs for those diagnosed with other diseases. Our findings show that the prevalence of Grades A and B after the age of 1 year for the Swod group is higher than that for the AS group but lower than that for the Sw/ood group. Another interesting finding shows that there was no difference (P > 0.05) in the prevalence of Grade C between the AS group and the Swod group only for males aged 3–14 years.

### The Sw/ood group without E-ALNs

Truthfully, the etiology of abdominal pain in children is very complex^14,15^. Clinicians often judge whether there are E-ALNs by US after excluding common acute abdomen conditions in children. When the examination results are negative, reasonable explanations include functional abdominal pain (FAP) and irritable bowel syndrome (IBS)^14^, which are characterized by chronic or recurrent gastrointestinal symptoms that are not explained by structural or biochemical abnormalities^16^. According to some studies^17,18^, abdominal pain is experienced weekly by as many as 13–38% of children and adolescents, while the cause (e.g., inflammatory and anatomic) is not found on evaluation for the vast majority of these patients^19^. Consequently, a considerable number of these children are diagnosed with FAP or IBS^14^. However, the etiology and pathogenesis of FAP and IBS are very complex and remain controversial^20–22^.

### Study limitations

First, this study is a retrospective, single-center study, which means that although there are a large number of subjects, errors may have been introduced in some aspects, and thus, it will be necessary to conduct a multicenter study in cooperation with other institutions in the future. Second, US examinations were performed by approximately ten sonographers on four types of US machines, which may affect the reproductivity of the data and thus influence the results of this research.

## Conclusions

In summary, to our knowledge, this study involved the largest number of subjects in the investigation of ALNs. Given that sonography has long been accepted as the preferred method for the diagnosis of E-ALNs^23,24^, it was meaningful to compile and analyze ALN data from > 200,000 subjects who had undergone sonography in the last five years. The threshold for determining abnormally sized ALNs proposed in 1993^3^ was considered reasonable in our study. However, considering the actual status of ALNs, this study recommends that the US diagnosis of ALNs be classified into Grades A-D to provide more valuable information to clinicians. Moreover, this study recommends that ALNs with an SAD ≥ 8 mm in children aged 1–3 years be considered abnormal and suggests that those with an SAD ≥ 5 mm in children aged 3–14 years have clinical implications.

## Materials and methods

### Study design and population

This study enrolled children from the Children's Hospital of Fudan University (National Children's Medical Center) between July 2014 and September 2019 who underwent ALN sonography for various indications. Relevant data included clinical symptoms and diagnosis, ALN grade according to US, sex (male, female), and age. In terms of clinical symptoms and diagnosis, the subjects were divided into three groups.

- Group 1 (AS group): the inclusion criterion was children who had undergone abdominal sonography for health and adolescent examinations, while the exclusion criterion was children with abdominal symptoms, respiratory symptoms and fever.
- Group 2 (Sw/ood group): the inclusion criterion was children who had undergone abdominal sonography due to a suspected E-ALNs diagnosis in the presence of either abdominal pain, nausea, vomiting, diarrhea, upper respiratory tract infection, or fever, while the exclusion criterion was children diagnosed with other diseases (e.g., appendicitis, intussusception, intestinal obstruction, lithiasis, hepatobiliary and pancreatic inflammatory diseases, ovarian torsion, and urogenital diseases) that could explain the symptoms after examination.
- Group 3 (Swod group) consisted of patients who were excluded from group 2.

In addition, children with tumors (including lymphoma and leukemia), tuberculosis, infectious mononucleosis, systemic lupus erythematosus (SLE), sarcoidosis, and immune diseases were excluded from this study. The study was approved by the Ethics Committee of the Children’s Hospital of Fudan University, China, and informed consent was obtained from the legal guardians of all subjects in accordance with the relevant guidelines and regulations.

### US examination

US was performed for all subjects with four types of machines, including a PHILIPS IU22, SIEMENS ACUSON Sequoia, PHILIPS EPIQ5, and GE VOLUSON 730 EXPERT, by experienced sonographers. First, the whole abdominal cavity was scanned with a low-frequency probe (e.g., a C5-2 for the PHILIPS IU22) to determine the presence of organic diseases. Then, the abdominal cavity was examined in different sections by using a high-frequency linear probe (e.g., a L12-5 for the PHILIPS IU22), with a focus on the periumbilical area, the right lower quadrant (RLQ), and the site of pain. Finally, the sizes of the largest 2 or 3 lymph nodes were recorded. All methods utilized for this study were performed in accordance with the relevant guidelines and regulations.

### Statistical analysis

Based on the SAD of the largest lymph node, the subjects were divided into four levels in this study: SAD ≥ 10 mm (Grade A); 8 mm ≤ SAD < 10 mm (Grade B); 5 mm ≤ SAD < 8 mm (Grade C); and SAD < 5 mm (Grade D, normal). Each group could be further divided either by sex, i.e., male and female, or by age range, i.e., ages 0–1, 1–3, 3–6, 6–10, 10–14, and 14–18 years.

Altogether, there were 206,775 total subjects in our study. Statistical analyses were performed using the statistical software SPSS (Version 20.0, which can be downloaded from https://www.ibm.com/analytics/SPSS-statistics-software) as follows:

1. The prevalence of the different grades of lymph nodes in different age-sex subgroups of groups 1–3 was calculated.
2. The Chi-squared test was applied to groups 1–3 to identify age-sex subgroups that differed significantly in the distribution of lymph nodes in general and then to find statistically significant differences between the AS and Sw/ood groups in the prevalence of Grades A, B, and C to identify the threshold for abnormally sized lymph nodes.
3. The distribution characteristics of the prevalence of Grades A, B, and C by age and sex in the three groups were analyzed and summarized.

All tests were performed at a two-sided significance level of 0.05.

## Data Availability

The datasets generated during the current study are available from the corresponding author on reasonable request.
