# Author Correction: Identifying threshold sizes for enlarged abdominal lymph nodes in different age ranges from about 200,000 individual’s data

**DOI:** 10.1038/s41598-021-88764-w

**Published:** 2021-04-21

**Authors:** Lili He, Yinghua Sun, Guoying Huang

**Affiliations:** 1 Department of Ultrasound, Children’s Hospital of Fudan University, Shanghai, 201102 People’s Republic of China; 2 Cardiovascular Center, Children’s Hospital of Fudan University, Shanghai, 201102 People’s Republic of China

Correction to: *Scientific Reports* 10.1038/s41598-021-81339-9, published online 19 January 2021

This Article contains errors in Reference 15, which is incorrectly given as:

Sanfilippo, F. M., Osullivan, T. A., Robinson, M., Oddy, W. H. & Olynyk, J. K. The relationship between abdominal pain and emotional wellbeing in children and adolescents in the Raine Study. *Sci. Rep.* **10**, 1646. 10.1038/s41598-020-58543-0 (2020).

The correct reference is listed below:

Ayonrinde, O. T. et al. The relationship between abdominal pain and emotional wellbeing in children and adolescents in the Raine Study. *Sci. Rep*. **10**, 1646. 10.1038/s41598-020-58543-0 (2020).

